# Efficacy and safety of high-intensity focused ultrasound cyclo-plasty in glaucoma

**DOI:** 10.1186/s12886-022-02622-5

**Published:** 2022-10-07

**Authors:** Di Chen, Xiu-Juan Guo, Shu-Ke Luo, Yan Lu, Xiu-Rong Tang

**Affiliations:** Department of Ophthalmology, Affiliated Foshan Hospital, Southern Medical University, Foshan, 528000 China

**Keywords:** Focused ultrasound cyclo-plasty, Glaucoma, Intraocular pressure

## Abstract

**Background:**

High-intensity focused ultrasound cyclo-plasty (UCP) is a recently developed glaucoma surgery. This study collected and analysed the clinical data of patients who underwent UCP to observe the efficacy and safety of this surgery in Chinese glaucoma patients.

**Methods:**

This was a retrospective study. The clinical data of all the patients who underwent UCP at Affiliated Foshan Hospital, Southern Medical University, were collected and analysed to evaluate the efficacy and safety of UCP. The main outcome measure was intraocular pressure, and the secondary outcome measures were best corrected visual acuity (logMAR) and complications.

**Results:**

Fifty-eight patients (61 eyes) were recruited for this study. IOP was dramatically decreased during the 12 months after UCP (*p*<0.05). The median IOP reduction during the 18 months post-procedure was more than 30%. The greatest reduction was at 1 month post-UCP (60.86%). The qualified success rate was more than 60% during the 18-month follow-up (Fig. 1). Poor follow up was found after 6-month post-UCP. The highest success rate was obtained at 7 days post-UCP (94.55%). No statistically significant decrease in BCVA in the vison group was observed at the follow-up visits, except for 1 day post-UCP. There was a statistically significant reduction in the use of IOP lowering medications during the 6 months post-UCP. No severe complications occurred.

**Conclusion:**

UCP is a safe and effective procedure for primary and refractive glaucoma at least during the 6 months post-UCP procedure. Studies with longer follow-up time and better follow up are needed to further confirm the long-term efficacy and safety of UCP in Chinese glaucoma patients.

**Supplementary Information:**

The online version contains supplementary material available at 10.1186/s12886-022-02622-5.

## Background

Glaucoma is the leading, irreversible blinding eye disease in the world. It is estimated that by 2020, approximately 79.6 million people will suffer from glaucoma [1]. Elevated intraocular pressure that is pathologically confirmed is an important risk factor for glaucoma. Reducing intraocular pressure (IOP) is the most effective strategy for delaying optic neuropathy and visual field loss in patients with glaucoma [2]. IOP lowering medications are first choice for IOP reduction in most types of glaucoma, but their use is not suitable for all glaucoma patients. Glaucoma surgery is still needed in patients with advanced glaucoma, IOP lowering medications intolerance and refractory glaucoma. Traditional glaucoma surgery includes glaucoma filtration surgery (trabeculectomy, glaucoma drainage valve implantation) and ciliary body surgery (transscleral ciliary cyclocryotherapy, transscleral cyclophotocoagulation, and endoscopic cyclophotocoagulation). Traditional glaucoma filtering surgery involves a large incision and is likely to causes bleb-related complications after filtering, such as a shallow anterior chamber, a bleb infection after filtering, drainage valve exposure and so on. Traditional ciliary body surgery is not the preferred surgical treatment because of likely detrimental effects and serious complications, such as vision loss and phthisis bulbi. This type of surgery is only suitable for end-stage glaucoma patients with very low vision (counting finger or less) or without vision. High-intensity focused ultrasound cyclo-plasty (UCP) is a recently developed cyclodestructive procedure that is recommended for patients with primary and refractive glaucoma because it does not require an incision and uses ultrasound energy to selectively coagulate the ciliary epithelium without damaging the surrounding tissue [3, 4].

UCP procedure has been highlighted in glaucoma research in Europe and India in past 5-10 years. A few reports were found among Chinese patients and UCP was just only applicated in refractive glaucoma or end stage glaucoma (with very low vision, such as less than counting finger) [5–10]. Few studied were performed BCVA comparison before and after UCP surgery [7, 10]. The efficacy and safety need to be investigated among Chinese glaucoma patients with better vision other than in end stage glaucoma.

This study collected and analysed the clinical data of the patients who underwent UCP at Affiliated Foshan Hospital, Southern Medical University, including primary and refractive glaucoma patients, to observe the efficacy and safety of this surgery.

## Methods

### Ethical approval

This study was approved by the Institutional Review Board of Affiliated Foshan Hospital, Southern Medical University. This was a retrospective study conducted at the Department of Ophthalmology, Affiliated Foshan Hospital, Southern Medical University. This study was performed in accordance with the Declaration of Helsinki and was registered on the Chinese Clinical Trial Registry (identifier: ChiCTR2200057547, 14/03/2022). All subjects recruited in this study signed informed consent forms.

### Study population

The clinical data of all the patients who underwent UCP at Affiliated Foshan Hospital, Southern Medical University (from 31 March 2020 to 26 August 2021) were collected.

### Clinical data collection

Main outcome measures: intraocular pressure (IOP). Secondary outcome measures: logMAR best corrected visual acuity (BCVA) and complications. Other clinical data that was collected: sex, age, lens status, glaucoma type, ocular surgery history, Numerical Rating Scale (NRS) pain scores, axis length, white-to-white, and IOP lowering medications types. Qualified success was defined as an IOP reduction ≥ 20% from the baseline value, with an IOP >5 mmHg, and no additional IOP lowering medications or glaucoma surgery.

Clinical data collection methods: The electronic medical record system of Affiliated Foshan Hospital, Southern Medical University was consulted, and the preoperative and postoperative follow-up information of the patients who underwent UCP was recorded.

### Surgical procedure

UCP procedures were performed with a second-generation probe EyeOP1 device (Eye Tech Care – France), a technique previously described [5, 6]. Standard parameters were selected (6 sectors when IOP was less than 21 mmHg, 8 sectors when IOP was from 21 mmHg to 40 mmHg, 10 sectors when IOP was greater than 40 mmHg, 21 MHz of frequency, duration of each shot of 8 s, and an interval of 20 s). Except for 1 eye that received systemic anaesthesia (because of obvious eye pain in the patient’s first eye when UCP was performed), the other eyes were under retrobulbar anaesthesia. All surgeries were performed by Dr. Xiujuan Guo. After UCP treatment, IOP lowering drops and topical glucocorticoids drops (1%prednisone) were prescribed in all patients. 1% atropine eye gel was just applied once in patients with obvious eye pain after UCP procedure.

### Statistical analysis

All clinical data were statistically analysed using IBM SPSS (SPSS, Chicago, IL, USA) statistical software version 26.0. Continuous variables are presented as the mean ± standard deviation (conforming to normal distribution) or presented as the median (P25, P75). Wilcoxon Signed Ranks Test was used to compare the baseline ocular parameters with those in each postoperative follow-up period. The IOP reduction (%) in each follow-up period was recorded by the quartile method. Chi square test was used to compare the success rate between the group with previous glaucoma surgery and the group without previous glaucoma surgery. In all statistical analyses, a p value less than 0.05 was considered statistically significant.

## Results

Fifty-eight patients (61 eyes) were recruited for this study, including 39 males and 19 females. The average age was 62.0±13.04 years. In 61 eyes, 56 were phakic, 4 were pseudophakic, and 1 was aphakic. In all the recruited eyes, 7 had primary open angel glaucoma (POAG), 17 had primary angle-closure glaucoma (PACG), 26 had neovascular glaucoma (NVG), and 11 had other types of secondary glaucoma. Of all 61 eyes, 47 eyes never underwent glaucoma surgery, and 14 eyes previously underwent glaucoma surgery. Three eyes underwent par plana vitrectomy (PPV) and silicone oil tamponade. Two eyes were treated with anti-vascular endothelial growth factor (VEGF). The pre-UCP IOP was less than 21 mmHg in 7 eyes, between 22 mmHg and 29 mmHg in 8 eyes, between 30 mmHg and 39 mmHg in 14 eyes, between 40 mmHg and 49 mmHg in 11 eyes, and equal to and/or greater than 50 mmHg in 21 eyes. The pre-UCP BCVA was no light perception (NLP) in 15 eyes, was less than 20/800 in 26 eyes, and equal to and/or more than 20/800 in 20 eyes (vision group). The maximum range of BCVA before UCP was 20/20. The average axial length was 23.43±1.05 mm, and the white-to-white was 11.72±0.54 mm. The average number of IOP lowering medications pre-UCP was 2.80±0.75. The median of NRS scores before UCP was 4. All the baseline parameters are shown in Table 1.

**Table 1:** Baseline demographic characteristics of eyes performed UCP (*n*=61)

Eyes (Right/Left)	26/35
Age (year)	62.09±13.04
Sex (n)	
Male	39
Female	19
Lens status (n)	
Phakic	54
Pseudophakic	6
Aphakic	1
Type of glaucoma (n)	
POAG	7
PACG	17
Secondary glaucoma	37
NVG	26
Other type of secondary glaucoma	11
Previous glaucoma surgery (n)	
None	47
Cyclocryotherapy	1
Trabeculectomy	11
LPI	1
Other ocular surgery (n)	
PPV and silicone oil tamponade	3
Anti-VEGF	2
Preoperative IOP (n)	
15-21mmHg	7
22–29 mmHg	8
30–39 mmHg	14
40–49 mmHg	11
≥50 mmHg	21
Preoperative visual acuity (n)	
No light perception	15
Light perception	9
Hand movement	15
Counting finger	2
≥ 20/800	20
Axial length (mm)	23.43±1.05
WTW (mm)	11.72±0.54
Pre-operative IOP lowering medications (*n*=61)	2.80±0.75
NRS pain scores (median, (P25,P75))	4 (0, 8)

Data on IOP at the 7 follow-up visits were collected (Table 2). IOP was dramatically decreased after UCP on postoperative day 1 and 7, and at 1, 3, 6 and 12 months (*p*<0.05). At 7 days, 1 month, 3 months,6 month, 12 months, and 18 months post-UCP, the median IOP was less than 21 mmHg.

**Table 2:** IOP and IOP reduction during each follow-up visit after UCP

	Number (n)		IOP (mmHg, median (P25, P75))			IOP reduction (%)		
		Preoperative IOP	Follow-up IOP	Z	*p*	P25	Median	P75
Day 1	61	40.80 (29.10, 53.05)	21.00 (14.50, 31.45)	-6.626	**<0.001**	24.86	39.13	58.44
Day 7	55	38.90 (28.00, 51.70)	10.60 (7.40, 24.60)	-6.334	**<0.001**	41.67	59.76	79.61
Month 1	40	36.50 (24.70, 48.08)	13.00 (8.63, 18.35)	-5.359	**<0.001**	45.23	60.86	74.55
Month 3	32	37.00 (26.50, 48.00)	14.35 (10.78, 24.90)	-4.366	**<0.001**	20.65	53.65	70.20
Month 6	32	37.00 (24.70, 48.08)	18.00 (11.95, 28.20)	-3.908	**<0.001**	9.95	33.48	68.95
Month 12	14	33.95 (22.23, 45.78)	16.15 (11.63, 35.93)	-2.132	**0.033**	6.25	30.60	67.99
Month 18	10	27.00 (24.28, 46.73)	20.20 (14.25, 41.48)	-1.125	0.260	-25.41	31.90	58.49

Bold values are statistically significant

The IOP reduction rate was calculated for each follow-up visit (Table 2). The median IOP reduction at all 7 follow-up visits was more than 30%. The greatest reduction was observed at the 1-month post-UCP visit (60.86%), then decreased to 53.65% at 3 months post-UCP, and remained at approximately 30% until 18 months postoperatively.

**Fig 1.**
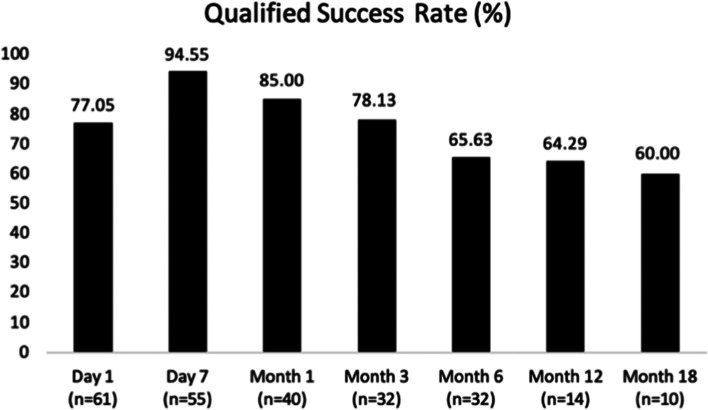
Qualified success rate during each follow-up visit after UCP (%)

The qualified success rate was more than 60% at all 7 follow-ups (Fig. 1). The highest success rate was obtained at 7 days post-UCP (94.55%) and then decreased over time. At 1 month and 3 months post-UCP, the success rates were 85.00% and 78.13%, respectively. At 6 months post-UCP, the success rate decreased to 65.63%.

Seven follow-up BCVA data points were collected (Table 3). No statistically BCVA deceased at post-UCP follow-up visits in vision group (with BCVA more than 20/800 pre-UCP), except for 1 day post-UCP. During the follow-up, the rate of patients with vision loss ≥2 lines increased. The possible causes of vision loss were the progression of a pre-existing cataract (*n*=6), transient hypotony (*n*=1), an astigmatism more than 3 diopters (*n*=1), diabetic retinopathy progression (*n*=2), possible glaucoma progression (*n*=2), pre-existing macular oedema resulting from central retinal vein occlusion progression under retinal photocoagulation and anti-VEGF treatment (*n*=1).

**Table 3:** LogMRA visual acuity at baseline and during each follow-up visit after UCP

	Follow-up number (n)	Preoperative BCVA	Follow-up BCVA	Z	*p*	Vision unchanged(n，%)	Vison loss 1 line(n，%)	Vision loss ≥2 lines(n，%)
Day 1	20	0.40 (0.13, 0.98)	0.55 (0.30, 0.90)	-1.985	**0.047**	12(60)	1(5)	7(35)
Day 7	18	0.50 (0.18, 1.03)	0.60 (0.38, 1.00)	-0.912	0.362	9(50)	3(16.7)	6(33.3)
Month 1	15	0.40 (0.10, 1.00)	0.70 (0.50, 1.10)	-1.486	0.137	8(53.3)	2(13.3)	5(33.3)
Month 3	15	0.40 (0.10, 1.00)	0.70 (0.40, 1.00)	-1.261	0.207	5(33.3)	2(13.3)	8(53.3)
Month 6	17	0.40 (0.10, 0.95)	0.70 (0.20, 1.00)	-1.462	0.144	7(41.2)	2(11.8)	8(47.1)
Month 12	9	0.80 (0.35, 1.15)	1.00 (0.45, 2.30)	-1.352	0.176	5(55.6)	0	4(44.4)
Month 18	5	0.80 (0.30, 1.00)	1.30 (0.20, 3.00)	-1.461	0.144	2(40)	0	3(60)

Bold values are statistically significant

At the 18-month follow-up, IOP lowering medications were significantly reduced on postoperative day 1 and 7, and at 1 month, 3 months, and 6 months post-UCP (Table 4). At 12 months and 18 months post-UCP, no significant difference was found in topical IOP lowering medications application (Table 4).

**Table 4:** IOP lowering medications (n) at baseline and during each follow-up visit after UCP

	Follow-up number (n)	Preoperative	Follow-up	Z	*p*
Day 1	61	3.00 (2.00,3.00)	2.00 (2.00,3.00)	-4.285	**<0.001**
Day 7	55	3.00 (2.00,3.00)	2.00 (1.00,3.00)	-5.028	**<0.001**
Month 1	40	3.00 (2.00,3.00)	0.50 (0.00, 3.00)	-4.827	**<0.001**
Month 3	32	3.00 (2.00,3.00)	1.50 (0.00, 2.75)	-3.754	**<0.001**
Month 6	32	3.00 (2.00,3.00)	2.00 (0.25, 3.00)	-3.306	**0.001**
Month 12	14	3.00 (2.00,3.00)	3.00 (2.00, 3.00)	-0.504	0.614
Month 18	10	2.50 (2.00, 3.00)	2.50 (1.00, 3.00)	-0.711	0.477

Bold values are statistically significant

Subgroup analysis with NVG eyes was performed (Table 5). IOP was dramatically decreased after UCP on postoperative day 1 and 7, and at 1, 3, 6 months in NVG group (*p*<0.05). The median IOP reduction at first 5 visits was more than 30%. The greatest reduction was observed at the 3-month post-UCP visit (72.02%). The qualified success rate was more than 70% at first 5 visits. The highest success rate was obtained at 7 days post-UCP (95.00%) and then decreased over time. At 1 month and 3 months post-UCP, the success rates were 93.33% and 90.00%, respectively. At 6 months post-UCP, the success rate decreased to 72.73%.IOP lowering medications were significantly reduced on postoperative day 1 and 7, and at 1 month, 3 months, and 6 months post-UCP(*p*<0.05). Very small sample size (<10) was found at 12 months and 18 months post-UCP. Analysis was not performed at 12 months and 18 months post-UCP visits because of small sample size. BCVA more than 20/800 was just found in 2 eyes. LogMAR BCVA of 1 eye improved from 1 to 0.7 at 12 months after UCP. Another eye was worsened from 0.3 to 0.7 at 12 months after UCP, due to diabetic retinopathy progression though under retinal photocoagulation and anti-VEGF treatment.

**Table 5:** Subgroup analysis for eyes with NVG (Median, (P25, P75), *p*)

	Follow-up number (n)		IOP (mmHg)			IOP reduction rate (%)			Success rate (%)		IOP-lowering medications (n)		
		Preoperative IOP	Follow-up IOP	Z value	*p*	P25	Median	P75		Preoperative	Follow-up	Z value	*p*
Day 1	26	49.65 (60.00, 57.45)	27.25 (20.25, 34.75)	-4.432	**<0.001**	24.93	37.91	65.17	84.62	3.00 (2.00, 3.00)	2.50 (2.00, 3.00)	-2.489	**0.013**
Day 7	20	49.65 (40.85, 59.15)	20.80 (9.48, 35.75)	-3.823	**<0.001**	36.35	49.35	81.13	95.00	3.00 (2.00, 3.00)	2.00 (1.25, 3.00)	-2.491	**0.013**
Month 1	15	42.00 (40.30, 60.00)	15.20 (9.00, 18.40)	-3.351	**0.001**	57.42	68.54	75.00	93.33	3.00 (2.00, 3.00)	2.00 (0.00, 3.00)	-2.584	**0.010**
Month 3	10	41.60 (40.68, 57.15)	11.35 (7.45, 24.80)	-2.803	**0.005**	53.32	72.02	85.35	90.00	3.00 (2.00, 3.25)	1.00 (0.00, 3.00)	-2.203	**0.028**
Month 6	11	42.30 (41.00, 60.00)	28.70 (8.90, 44.80)	-2.490	**0.013**	17.97	52.17	78.81	72.73	3.00 (2.00, 3.00)	2.00 (0.00, 3.00)	-2.325	**0.020**

Bold values are statistically significant

Subgroup analysis was performed in eyes with and without previous glaucoma surgery, separately (Table 6). IOP was dramatically decreased after UCP on postoperative day 1 and 7, and at 1 month in these 2 groups. IOP lowering medications were also significantly decreased in the 2 groups. No significant difference was found between the group with and without previous glaucoma surgery in IOP reduction and success rate.

**Table 6:** Subgroup analysis for eyes with or without previous glaucoma surgery (Median, (P25, P75), *p*)

	(n)	IOP (mmHg)				IOP reduction rate (%)			Success rate (%)			IOP lowering medications (n)			
		Preoperative IOP	Follow-up IOP	Z	*p*	Median (P25, P75)	*Z	*p		*χ^2^	*p	Pre-UCP	Follow-up	Z	*p*
Group A															
Day 1	13	28.00 (21.80, 39.05)	17.00 (12.50, 23.00)	-2.621	**0.009**	40.53 (1.36, 60.54)	-1.153	0.249	69.23	0.310	0.578	3(2, 3)	2(1.5, 2.5)	-2.232	**0.026**
Day 7	13	28.00 (21.80, 39.05)	9.30 (7.50, 11.15)	-3.180	**0.001**	67.14 (55.83, 71.82)	-0.175	0.861	100	0.567	0.452	3(2, 3)	2 (0.5, 2)	-2.549	**0.011**
Month 1	9	25.00 (21.80, 41.65)	9.70 (7.30, 16.55)	-2.666	**0.008**	65.04 (50.91, 69.76)	-0.178	0.859	100	0.812	0.367	3 (2, 3)	1(0, 3)	-2.414	**0.016**
Group B															
Day 1	48	41.65 (33.75, 56.15)	24.50 (15.18, 32.18)	-5.958	**<0.001**	38.73 (26.25, 58.01)	-	-	81.25	-	-	3 (2, 3)	2 (2,3)	-3.684	**<0.001**
Day 7	42	41.10 (32.48, 56.30)	11.90 (7.25, 29.30)	-5.511	**<0.001**	58.62 (39.82, 80.96)	-	-	88.10	-	-	3 (2, 3)	2 (1,3)	-4.351	**<0.001**
Month 1	31	38.00 (30.20, 51.50)	13.80 (9.00, 19.00)	-4.679	**<0.001**	60.54 (37.67, 75.00)	-	-	80.65	-	-	3 (2, 3)	0 (0, 3)	-4.237	**<0.001**

Bold values are statistically significant

Group A: Eyes with previous glaucoma surgery; Group B: Eyes without previous glaucoma surgery

*Comparison for the group A and group B

In all the eyes that underwent UCP, no severe complications occurred (Table 7). All eyes had transient mild conjunctival hyperaemia, which recovered within 1 week to 1 month. Scleral impression was found in 2 eyes and recovered in 2-3 months. Three eyes had transient hypotony (IOP=5 mmHg), which recovered after IOP lowering medications were stopped. One eye had choroidal detachment and recovered after treatment with systemic and topical steroids and cycloplegic agents. Twenty-one eyes had mild anterior chamber inflammation, which recovered at 1-2 weeks. One eye had mydriasis. One eye had obvious astigmatism. No corneal edeme, superficial punctate keratitis, hyphema and subconjunctival hemorrhage were there.

**Table 7:** Therapeutic outcome of intra- and post-operative complications

Complications	Number (n)	Therapeutic outcome
Conjunctival Hyperemia	61	Recovery within 1 week to 1 month
Scleral impression	2	Recovery in 2-3months
Hypotony	3	Recovery after IOP lowering medications stopped
Choroidal detachment	1	Recovery after treatment in 1 month
Mydriasis	1	Unrecovered
Anterior chamber inflammation	21	Recovery in 1-2 weeks
Induced astigmatism	1	Unrecovered

## Discussion

This study retrospectively investigated the efficacy and safety of high-intensity focused ultrasound cyclo-plasty. At 7 post-UCP follow-up visits (ranging from 1 day to 18 months), IOP, IOP reduction rate, qualified success rate, logMAR BCVA, complications, and topical IOP lowering medications application were analysed. IOP was dramatically decreased during 6 visits (ranging from 1 day to 12 months), except for 18 months after UCP. The median IOP reduction during the 18 months post-UCP was more than 30%. The greatest reduction rate was at 1 month post-UCP (60.86%) and remained at approximately 30% until 18 months. The qualified success rate was more than 60% at 18 months post procedure. No statistically significant reduction in BCVA in the vision group (eyes with BCVA more than 20/800) was observed at the follow-up visits, except for 1 day post-UCP. There was a statistically significant reduction in topical IOP lowering medications application during the 6 months post-UCP. In subgroup analysis, IOP was dramatically decreased in NVG groups. BCVA was not impaired because of the procedure. No significant difference was found between the group with and without previous glaucoma surgery in IOP reduction and success rate.

The main mechanism of IOP reduction after UCP includes the following: coagulation at the ciliary epithelium, resulting in a reduction in aqueous humour secretion; an increase in the aqueous outflow through the supraciliary space, suprachoroidal space and from the thinned sclera to the subconjunctival area; and an increase in the aqueous outflow through the trabecular meshwork [3, 7–9]. Reduction of aqueous humour secretion is the leading cause of decreased IOP. Several studies described a significant decrease in IOP from 1 day to 6 months after UCP [8, 10–15]. Some studies found that IOP decreased during the 12 months after UCP [16–19]. Those studies with a longer follow-up period found that IOP decreased during the 24 months after UCP [20–22]. In the current study, IOP decreased during the 12 months post-UCP and increased at 18 months. In an earlier study, Graber et al. found no significant reduction in IOP in PACG patients after UCP (*n*=7); the probable causes may include the small sample size and the method (with the first-generation probes and activation of each transducer for 6 seconds) in the study [23].

Zhou et al. found a 20% reduction in IOP at the 12-month follow-up, and the greatest IOP reduction was observed at 3 months after UCP (42%) [8]. Graber et al. described a lower IOP reduction (2%-21%) during the 12 months post-operation [24]. Luo et al. reported the greatest reduction in IOP of more than 40% (achieved at 1 month post-UCP) and Wang et al. reported a more than 30% reduction IOP during the 6 months post-UCP (achieved at 3 months post-UCP) [11, 12]. During the 24 months after UCP, similar results were also achieved, and the greatest IOP reduction was achieved at 1 day and at 7 days postprocedure [21, 22]. Twelve months after UCP, the greatest IOP reduction of 38%-67% was achieved, and the greatest reduction was observed at 1 day and 7 days postprocedure, with a more than 30% reduction in IOP at the time of the last visit [14, 17–19]. Most of the previous studies mentioned above reported that the greatest reduction in IOP was approximately 40%-60%, and the reduction was achieved at an early follow-up time (from 1 day to 3 months). We found a 30%-60% reduction in IOP during the 18 months post-UCP, and the greatest reduction in IOP was observed at 1 month, which was similar to these studies. Giannaccare et al. found that the reduction in IOP was 22%-32%, and the greatest reduction in IOP was achieved at 12 months. This time point is very different from other studies and may have resulted in additional IOP lowering medications applications as long as the follow-up time was reached [6].

The success rate will decrease over time after UCP. Some studies found a success rate of more than 60% until 6 months after UCP [11, 13, 23]. Wang et al. described that at 6 months after UCP, the success rate was 76% [12]. Giannaccare found a 51% success rate at 12 months postprocedure [25]. Zhou et al. found a 42%-92% success rate at 12 months postprocedure [8]. Figus et al. and Wang et al. described a more than 70% success rate at 24 months after UCP [10, 21]. Leshno et al. found an 87% success rate at 24 months after UCP [22]. We found a 60%-94% success rate during the 18-month follow-up, and a 60% success rate was achieved at 18 months postprocedure, which was similar to previous studies. However, in a few studies, a lower success rate was achieved [13, 24].

Zhou et al. found that the number of topical IOP lowering medications applications decreased from 1 day to 3 months after UCP [8]. Luo et al. and Liu et al. found that topical IOP lowering medications applications decreased at 6 months after UCP [11, 13]. The results of applied topical IOP lowering medications in our study were consistent with the above 2 studies. Several studies described a decrease in the application of topical IOP lowering medications over 12 months [4, 16, 19]. Decreased topical IOP lowering medications applications during the 24 months post-UCP were described by Giannaccare et al. [20]. However, in a few studies, the number of topical IOP lowering medications applications did not change [21, 22].

Figus et al. found unchanged vision in 44%-78% of patients at 24 months post-UCP, and vision loss more than 2 lines occurred in 9%-20% of patients whose logMAR vision was equal to or less than 1 [21]. Most studies found no significant BCVA change after UCP [4, 14–16, 18, 25]. In these studies, unchanged vision was found in 40%-60% of the eyes at 18 months postprocedure, and vision loss of more than 2 lines occurred in 33-60% of the eyes. The most common cause of vision loss was pre-existing cataracts or other fundus disease progression, which alerts us that regular follow-up visits and positive treatment for primary manifestations are necessary after UCP.

Ultrasound coagulation at the ciliary epithelium selectively will not destroy the surrounding tissue, therefore avoiding severe vision-threatening complications. No severe complications were reported in most studies after UCP. The most common complication was conjunctival hyperaemia after the operation. Other mild complications included anterior chamber reaction, subconjunctival haemorrhage, superficial punctate keratitis, mydriasis, posterior synechiae, focal scleral thinning, and scleral marks. More severe complications, such as hyphema, transient hypotony, choroidal detachment, and macular oedema, have also been reported [4, 10, 13, 19]. Wang et al. reported that phthisis bulbi, a severe complication, occurred in 1 patient after UCP [12]. In the present study, 1 eye had choroidal detachment post-UCP, which recovered after treatment.

Besides UCP, micropulse transscleral cyclophotocoagulation (MP-CPC) is an another novel ciliary body surgery in glaucoma treatment with good efficacy and safety [26–29]. The mechanism of MP-CPC was similar to UCP, also selective coagulation at the ciliary epithelium, resulting in a reduction in aqueous humour secretion, except for using laser instead of ultrasound energy. Increased uveoscleral outflow was also found after MP-CPC treatment [30]. It would be interesting to compared UCP and MP-CPC in glaucoma treatment in the future.

There are several limitations in this study. This was a retrospective study. The loss to follow-up rate was higher than that of a prospective design study, especially at the 12-month post-UCP visit and thereafter. Therefore, a small sample of patients were included in these follow-up visits. A main cause of loss to follow-up was that patients with low vision refused to follow-up when eye pain was relieved after UCP. These patients care more about the symptoms of eye pain than their IOP value and visual acuity. Another reason was patients went to a nearby hospital for ocular examinations，especially when the COVID-19 epidemic was more serious.

In conclusion, UCP is a safe and effective procedure for primary and refractive glaucoma at least during the 6 months post-UCP procedure. Studies with longer follow-up time and better follow up are needed to further confirm the long-term efficacy and safety of UCP in Chinese glaucoma patients.

## Supplementary Information

**Additional file 1.**

## Data Availability

All data generated or analysed during this study are included in this published article [and its supplementary information files].

## Abbreviations

IOP: Intraocular pressure
UCP: Ultrasound cyclo-plasty
POAG: Open angel glaucoma
PACG: Primary angle-closure glaucoma
NVG: Neovascular glaucoma
PPV: Par plana vitrectomy
BCVA: Best corrected visual acuity
VEGF: Vascular endothelial growth factor
NLP: No light perception
NRS: Numerical Rating Scale
MP-CPC: Micropulse transscleral cyclophotocoagulation

## References

[1] Quigley HA, Broman AT (2006). The number of people with glaucoma worldwide in 2010 and 2020. Br J Ophthalmol..

[2] Sihota R, Angmo D, Ramaswamy D, Dada T (2018). Simplifying "target" intraocular pressure for different stages of primary open-angle glaucoma and primary angle-closure glaucoma. Indian J Ophthalmol.

[3] Aptel F, Charrel T, Lafon C, Romano F, Chapelon JY, Blumen-Ohana E, Nordmann JP, Denis P (2011). Miniaturized high-intensity focused ultrasound device in patients with glaucoma: a clinical pilot study. Invest Ophthalmol Vis Sci..

[4] Torky MA, Alzafiri YA, Abdelhameed AG, Awad EA (2021). Phaco-UCP; combined phacoemulsification and ultrasound ciliary plasty versus phacoemulsification alone for management of coexisting cataract and open angle glaucoma: a randomized clinical trial. BMC Ophthalmol..

[5] Longfang Z, Die H, Jie L, Yameng L, Mingyuan L, Xiaojing P (2022). Efficacy and safety of single Ultrasound Cyclo-Plasty to treat refractory glaucoma: Results at 1 year. Eur J Ophthalmol..

[6] Wang T, Wang R, Su Y, Li N (2021). Ultrasound cyclo plasty for the management of refractory glaucoma in chinese patients: a before-after study. Int Ophthalmol..

[7] Luo Q, Xue W, Wang Y, Chen B, Wang S, Dong Y, et al. Ultrasound cycloplasty in Chinese glaucoma patients: Results of a 6-month prospective clinical study. Ophthalmic Res. 2021. 10.1159/000515013. 10.1159/000515013 33540418

[8] Ruixue W, Tao W, Ning L (2020). A comparative study between ultrasound cycloplasty and cyclocryotherapy for the treatment of neovascular glaucoma. J Ophthalmol.

[9] Liu HT, Zhang Q, Jiang ZX, Xu YX, Wan QQ, Tao LM (2020). Efficacy and safety of high-dose ultrasound cyclo-plasty procedure in refractory glaucoma. Int J Ophthalmol..

[10] Yu Q, Liang Y, Ji F, Yuan Z (2020). Comparison of ultrasound cycloplasty and transscleral cyclophotocoagulation for refractory glaucoma in Chinese population. BMC Ophthalmol..

[11] Aptel F, Denis P, Rouland JF, Renard JP, Bron A (2016). Multicenter clinical trial of high-intensity focused ultrasound treatment in glaucoma patients without previous filtering surgery. Acta Ophthalmol..

[12] Giannaccare G, Sebastiani S, Campos EC. ultrasound cyclo plasty in eyes with glaucoma. J Vis Exp. 2018;131. 10.3791/56192. 10.3791/56192 PMC5908701 29443031

[13] Mastropasqua R, Agnifili L, Fasanella V, Toto L, Brescia L, Di Staso S, Doronzo E, Marchini G (2016). Uveo-scleral outflow pathways after ultrasonic cyclocoagulation in refractory glaucoma: an anterior segment optical coherence tomography and in vivo confocal study. Br J Ophthalmol..

[14] Aptel F, Begle A, Razavi A, Romano F, Charrel T, Chapelon JY, Denis P, Lafon C (2014). Short- and long-term effects on the ciliary body and the aqueous outflow pathways of high-intensity focused ultrasound cyclocoagulation. Ultrasound Med Biol..

[15] Pellegrini M, Sebastiani S, Giannaccare G, Campos EC (2019). Intraocular inflammation after Ultrasound Cyclo Plasty for the treatment of glaucoma. Int J Ophthalmol..

[16] Giannaccare G, Vagge A, Gizzi C, Bagnis A, Sebastiani S, Del Noce C, Fresina M, Traverso CE, Campos EC (2017). High-intensity focused ultrasound treatment in patients with refractory glaucoma. Graefes Arch Clin Exp Ophthalmol..

[17] Torky MA, Al Zafiri YA, Hagras SM, Khattab AM, Bassiouny RM, Mokbel TH (2019). Safety and efficacy of ultrasound ciliary plasty as a primary intervention in glaucoma patients. Int J Ophthalmol..

[18] Deb-Joardar N, Reddy KP (2018). Application of high intensity focused ultrasound for treatment of open-angle glaucoma in Indian patients. Indian J Ophthalmol.

[19] Morais Sarmento T, Figueiredo R, Garrido J, Passos I, Rebelo AL, Candeias A (2021). Ultrasonic circular cyclocoagulation prospective safety and effectiveness study. Int Ophthalmol..

[20] Giannaccare G, Pellegrini M, Bernabei F, Urbini L, Bergamini F, Ferro Desideri L, Bagnis A, Biagini F, Cassottana P, Del Noce C, Carnevali A, Scorcia V, Traverso CE, Vagge A (2021). A 2-year prospective multicenter study of ultrasound cyclo plasty for glaucoma. Sci Rep..

[21] Figus M, Posarelli C, Nardi M, Stalmans I, Vandewalle E, Melamed S, et al. Ultrasound Cyclo Plasty for Treatment of Surgery-Naive Open-Angle Glaucoma Patients: A Prospective, Multicenter, 2-Year Follow-Up Trial. J Clin Med. 2021;10(21). 10.3390/jcm10214982. 10.3390/jcm10214982 PMC8584324 34768500

[22] Leshno A, Rubinstein Y, Singer R, Sher I, Rotenstreich Y, Melamed S, Skaat A (2020). High-intensity focused ultrasound treatment in moderate glaucoma patients: results of a 2-year prospective clinical trial. J Glaucoma..

[23] Graber M, Khoueir Z, Beauchet A, Benhatchi N, Hammoud S, Lachkar Y (2017). High intensity focused ultrasound as first line treatment in patients with chronic angle closure glaucoma at risk for malignant glaucoma. J Fr Ophtalmol..

[24] Graber M, Rothschild PR, Khoueir Z, Bluwol E, Benhatchi N, Lachkar Y (2018). High intensity focused ultrasound cyclodestruction versus cyclodiode treatment of refractory glaucoma: A retrospective comparative study. J Fr Ophtalmol..

[25] Giannaccare G, Vagge A, Sebastiani S, Urbini LE, Corazza P, Pellegrini M, Carmassi L, Bergamini F, Traverso CE, Campos EC (2019). Ultrasound cyclo-plasty in patients with glaucoma: 1-year results from a multicentre prospective study. Ophthalmic Res..

[26] Huth A, Viestenz A (2022). Micropulse cyclophotocoagulation lowers the intraocular pressure: half year results. Ophthalmologe..

[27] Souissi S, Le Mer Y, Metge F, Portmann A, Baudouin C, Labbe A, Hamard P (2021). An update on continuous-wave cyclophotocoagulation (CW-CPC) and micropulse transscleral laser treatment (MP-TLT) for adult and paediatric refractory glaucoma. Acta Ophthalmol..

[28] Kotula MA, Paust K, Wirdemann A, Msigomba E, Burusu L. Glaucoma treatment by transscleral cyclophotocoagulation in micropulse technology in a low-income setting. Ophthalmologie. 2022. 10.1007/s00347-022-01668-6. 10.1007/s00347-022-01668-6 35925334

[29] Sarrafpour S, Saleh D, Ayoub S, Radcliffe NM (2019). Micropulse Transscleral Cyclophotocoagulation: A Look at Long-Term Effectiveness and Outcomes. Ophthalmol Glaucoma..

[30] Nemoto H, Honjo M, Okamoto M, Sugimoto K, Aihara M (2022). Potential Mechanisms of Intraocular Pressure Reduction by Micropulse Transscleral Cyclophotocoagulation in Rabbit Eyes. Invest Ophthalmol Vis Sci..

