# Supplementary material for: Efficacy and safety of high-intensity focused ultrasound cyclo-plasty in glaucoma
Source: BMC Ophthalmol. 2022 Oct 7;22:401. doi: 10.1186/s12886-022-02622-5 (PMC9542456; doi:10.1186/s12886-022-02622-5)
Supplement: Supplementary file 1 — Additional file 1. [file 12886_2022_2622_MOESM1_ESM.pdf]

| Age | Axial length (mm) | WTW(mm) | Pre-UCP AGMs(n) | NRS pain scores | Pre-UCP BCVA | matched D 1 post-UCP |
|---|---|---|---|---|---|---|
| 67 | 24.21 | 11.90 | 3.00 | 9 | 0.80 | |
| 55 | 24.13 | 11.96 | 2.00 | 8 | 1.00 | |
| 45 | 23.93 | 10.87 | 3.00 | 8 | 0.80 | |
| 76 | 23.52 | 11.78 | 4.00 | 7 | 0.10 | |
| 82 | 22.99 | 12.88 | 1.00 | 8 | 0.90 | |
| 67 | 24.31 | 12.18 | 2.00 | 8 | 0.40 | |
| 48 | 23.54 | 11.00 | 2.00 | 0 | 0.00 | |
| 56 | 23.78 | 12.00 | 3.00 | 3 | 1.10 | |
| 46 | 25.52 | 12.00 | 3.00 | 4 | 0.00 | |
| 45 | 22.04 | 11.60 | 3.00 | 6 | 0.20 | |
| 74 | 23.03 | 12.50 | 2.00 | 0 | 0.40 | |
| 79 | 23.58 | 12.00 | 2.00 | 2 | 0.40 | |
| 49 | 24.22 | 10.90 | 3.00 | 8 | 0.30 | |
| 79 | 24.21 | 11.00 | 3.00 | 8 | 1.20 | |
| 52 | 24.27 | 12.00 | 2.00 | 6 | 0.60 | |
| 75 | 23.59 | 11.10 | 3.00 | 7 | 1.60 | |
| 75 | 24.15 | 11.50 | 4.00 | 2 | 0.10 | |
| 72 | 23.07 | 11.70 | 2.00 | 7 | 0.40 | |
| 48 | 23.17 | 12.20 | 2.00 | 8 | 0.10 | |
| 71 | 24.18 | 12.09 | 2.00 | 0 | 1.20 | |
| 50 | 21.83 | 10.90 | 3.00 | 1 | | |
| 48 | 21.8 | 11.00 | 2.00 | 4 | | |
| 63 | 21.49 | 11.40 | 2.00 | 6 | | |
| 53 | 22.94 | 11.30 | 3.00 | 8 | | |
| 57 | 23.73 | 11.20 | 3.00 | 7 | | |
| 51 | 26.5 | 12.60 | 4.00 | 0 | | |
| 74 | 23.21 | 11.90 | 2.00 | 0 | | |
| 51 | 23.09 | 11.60 | 3.00 | 5 | | |
| 70 | 23.21 | 11.90 | 2.00 | 0 | | |
| 60 | 23.09 | 11.60 | 3.00 | 0 | | |
| 70 | 23.32 | 11.40 | 1.00 | 0 | | |
| 67 | 23.35 | 11.90 | 3.00 | 2 | | |
| 58 | 23.57 | 11.70 | 3.00 | 0 | | |
| 67 | 22.04 | 11.30 | 3.00 | 0 | | |
| 76 | 23.92 | 11.80 | 4.00 | 7 | | |
| 53 | 24.02 | 12.50 | 3.00 | 0 | | |
| 77 | 23.07 | 11.04 | 3.00 | 8 | | |
| 79 | 24.51 | 12.00 | 3.00 | 0 | | |
| 48 | 22.29 | 12.10 | 2.00 | 9 | | |
| 48 | 23.74 | 12.10 | 2.00 | 0 | | |
| 71 | 23.67 | 11.70 | 3.00 | 0 | | |
| 57 | 22.97 | 11.50 | 3.00 | 8 | | |
| 76 | 23.84 | 12.20 | 3.00 | 8 | | |
| 69 | 23.6 | 12.70 | 3.00 | 7 | | |
| 57 | 21.96 | 11.50 | 3.00 | 6 | | |
| 66 | 23.29 | 11.40 | 2.00 | 2 | | |
| 60 | 23.37 | 11.50 | 3.00 | 6 | | |
| 53 | 23.22 | 11.84 | 4.00 | 3 | | |
| 85 | 24.5 | 12.60 | 3.00 | 0 | | |
| 65 | 23.88 | 12.40 | 4.00 | 9 | | |
| 16 | 22.91 | 11.33 | 4.00 | 7 | | |

| | | | | |
|---|---|---|---|---|
| 52 | 24.57 | 12.90 | 4.00 | 10 |
| 48 | 23.02 | 11.20 | 2.00 | 7 |
| 76 | 25.92 | 12.40 | 4.00 | 8 |
| 70 | 22.04 | 10.80 | 3.00 | 0 |
| 72 | 22.08 | 11.10 | 3.00 | 0 |
| 53 | 24.37 | 12.10 | 3.00 | 2 |
| 74 | 22.05 | 11.10 | 3.00 | 2 |
| | 21.79 | 11.30 | 3.00 | 3 |
| | 20.93 | 11.20 | 4.00 | 3 |
| | 24.95 | 12.00 | 2.00 | 4 |

| BCVA D 1 post-UCP | Pre-UCP BCVA matched D 7 post-UCP | BCVA D 7 post-UCP | Pre-UCP BCVA matched M 1 post-UCP |
|---|---|---|---|
| 0.80 | 0.80 | 1.00 | 0.80 |
| 0.90 | 1.00 | 0.50 | 1.00 |
| 0.80 | 0.80 | 0.70 | 0.80 |
| 0.20 | 0.90 | 0.30 | 0.00 |
| 0.90 | 0.00 | 0.20 | 1.10 |
| 0.30 | 1.10 | 1.00 | 0.00 |
| 0.20 | 0.00 | -0.10 | 0.20 |
| 1.00 | 0.20 | 0.00 | 0.40 |
| 0.40 | 0.40 | 0.50 | 0.40 |
| 0.20 | 0.40 | 0.70 | 0.30 |
| 0.40 | 0.30 | 0.40 | 0.60 |
| 0.30 | 1.20 | 1.20 | 1.60 |
| 0.50 | 0.60 | 0.50 | 0.10 |
| 1.20 | 1.60 | 1.80 | 0.10 |
| 0.60 | 0.10 | 0.50 | 1.20 |
| 1.80 | 0.40 | 1.00 | |
| 0.40 | 0.10 | 0.70 | |
| 0.80 | 1.20 | 1.20 | |
| 0.30 | | | |
| 1.10 | | | |

| BCVA M 1 post-UCP | Pre-UCP BCVA matched M 3 post-UCP | BCVA M 3 post-UCP | Pre-UCP BCVA matched M6 post-UCP |
|---|---|---|---|
| 1.00 | 0.80 | 1.00 | 0.80 |
| 0.50 | 1.00 | 0.50 | 1.00 |
| 0.70 | 0.80 | 0.70 | 0.80 |
| 0.60 | 0.10 | 0.40 | 0.10 |
| 1.00 | 0.00 | 0.20 | 0.90 |
| 0.10 | 1.10 | 1.00 | 0.40 |
| 0.00 | 0.00 | 0.00 | 0.00 |
| 0.50 | 0.20 | 0.00 | 1.10 |
| 1.10 | 0.40 | 0.70 | 0.00 |
| 1.30 | 0.40 | 0.50 | 0.20 |
| 0.50 | 0.30 | 1.00 | 0.40 |
| 1.80 | 1.20 | 0.70 | 0.40 |
| 1.50 | 1.60 | 1.80 | 0.30 |
| 0.70 | 0.10 | 1.00 | 1.20 |
| 1.00 | 0.10 | 0.40 | 1.60 |
| | | | 0.10 |
| | | | 0.10 |

| BCVA M 6 post-UCP | Pre-UCP BCVA matched M 12 post-UCP | BCVA M 12 post-UCP | Pre-UCP BCVA matched M18 post-UCP |
|---|---|---|---|
| 1.00 | 0.80 | 1.30 | 0.80 |
| 1.00 | 1.00 | 0.70 | 0.80 |
| 0.70 | 0.80 | 3.00 | 0.20 |
| 0.20 | 1.10 | 1.00 | 0.40 |
| 3.00 | 0.00 | 0.00 | 1.20 |
| 1.00 | 0.40 | 0.20 | |
| 0.20 | 0.30 | 0.70 | |
| 0.70 | 1.20 | 3.00 | |
| 0.20 | 1.60 | 1.60 | |
| 0.00 | | | |
| 0.70 | | | |
| 0.20 | | | |
| 1.00 | | | |
| 0.40 | | | |
| 1.80 | | | |
| 0.30 | | | |
| 0.50 | | | |

| BCVA M 18 post-UCP | Pre-UCP IOP matched D 1 afer UCP | IOP D 1 after UCP | IOP reduction D 1 after UCP(%) |
|---|---|---|---|
| 1.30 | 60.00 | 34.10 | 43.17 |
| 3.00 | 56.00 | 15.70 | 71.96 |
| 0.20 | 56.20 | 42.30 | 24.73 |
| 0.20 | 60.00 | 31.80 | 47.00 |
| 3.00 | 50.00 | 32.40 | 35.20 |
| | 60.00 | 11.20 | 81.33 |
| | 24.60 | 25.30 | -2.85 |
| | 40.80 | 24.70 | 39.46 |
| | 42.30 | 25.50 | 39.72 |
| | 54.40 | 28.30 | 47.98 |
| | 24.60 | 17.30 | 29.67 |
| | 38.90 | 8.80 | 77.38 |
| | 60.00 | 50.00 | 16.67 |
| | 60.00 | 30.90 | 48.50 |
| | 41.20 | 37.10 | 9.95 |
| | 41.30 | 16.80 | 59.32 |
| | 30.90 | 27.20 | 11.97 |
| | 56.60 | 18.00 | 68.20 |
| | 51.70 | 46.10 | 10.83 |
| | 23.30 | 24.30 | -4.29 |
| | 33.30 | 37.00 | -11.11 |
| | 37.00 | 15.70 | 57.57 |
| | 41.10 | 15.00 | 63.50 |
| | 51.50 | 31.10 | 39.61 |
| | 49.30 | 32.30 | 34.48 |
| | 17.30 | 20.30 | -17.34 |
| | 18.00 | 13.30 | 26.11 |
| | 41.00 | 24.70 | 39.76 |
| | 17.30 | 11.00 | 36.42 |
| | 20.00 | 13.30 | 33.50 |
| | 26.00 | 17.50 | 32.69 |
| | 32.30 | 6.30 | 80.50 |
| | 30.30 | 6.80 | 77.56 |
| | 19.00 | 11.30 | 40.53 |
| | 42.00 | 29.00 | 30.95 |
| | 28.00 | 21.00 | 25.00 |
| | 60.00 | 41.00 | 31.67 |
| | 28.00 | 13.70 | 51.07 |
| | 51.00 | 17.50 | 65.69 |
| | 23.00 | 14.00 | 39.13 |
| | 15.00 | 11.00 | 26.67 |
| | 48.00 | 30.00 | 37.50 |
| | 60.00 | 38.50 | 35.83 |
| | 40.30 | 22.70 | 43.67 |
| | 57.00 | 14.00 | 75.44 |
| | 37.00 | 13.20 | 64.32 |
| | 60.00 | 21.00 | 65.00 |
| | 37.00 | 17.00 | 54.05 |
| | 18.00 | 17.00 | 5.56 |
| | 60.00 | 60.00 | 0.00 |
| | 47.30 | 30.70 | 35.10 |

| | | |
|---|---|---|
| 60.00 | 37.00 | 38.33 |
| 50.00 | 6.30 | 87.40 |
| 51.00 | 47.00 | 7.84 |
| 25.00 | 20.70 | 17.20 |
| 33.00 | 16.30 | 50.61 |
| 36.00 | 29.00 | 19.44 |
| 30.20 | 8.00 | 73.51 |
| 38.00 | 15.00 | 60.53 |
| 36.70 | 18.30 | 50.14 |
| 36.00 | 34.00 | 5.56 |

| Pre-UCP IOP matched D 7 post-UCP | IOP D 7after UCP | IOP reduction D 7 after UCP(%) | Pre-UCP IOP  matched M 1 afer UCP |
|---|---|---|---|
| 60.00 | 45.00 | 25.00 | 60.00 |
| 56.20 | 33.80 | 39.86 | 60.00 |
| 60.00 | 38.20 | 36.33 | 60.00 |
| 50.00 | 32.00 | 36.00 | 24.60 |
| 60.00 | 10.00 | 83.33 | 40.80 |
| 24.60 | 7.30 | 70.33 | 42.30 |
| 40.80 | 24.60 | 39.71 | 24.60 |
| 42.30 | 7.10 | 83.22 | 38.90 |
| 54.40 | 15.40 | 71.69 | 60.00 |
| 24.60 | 10.80 | 56.10 | 41.20 |
| 38.90 | 6.40 | 83.55 | 30.90 |
| 60.00 | 35.00 | 41.67 | 56.60 |
| 60.00 | 25.80 | 57.00 | 23.30 |
| 41.20 | 18.30 | 55.58 | 33.30 |
| 41.30 | 8.50 | 79.42 | 51.50 |
| 30.90 | 5.00 | 83.82 | 17.30 |
| 56.60 | 36.00 | 36.40 | 18.00 |
| 51.70 | 28.40 | 45.07 | 41.00 |
| 23.30 | 7.30 | 68.67 | 17.30 |
| 33.30 | 13.40 | 59.76 | 20.00 |
| 37.00 | 11.30 | 69.46 | 26.00 |
| 41.10 | 11.00 | 73.24 | 32.30 |
| 51.50 | 10.50 | 79.61 | 30.30 |
| 49.30 | 39.00 | 20.89 | 19.00 |
| 17.30 | 10.30 | 40.46 | 42.00 |
| 18.00 | 9.00 | 50.00 | 23.00 |
| 41.00 | 16.50 | 59.76 | 15.00 |
| 17.30 | 12.60 | 27.17 | 40.30 |
| 20.00 | 8.40 | 58.00 | 57.00 |
| 26.00 | 10.60 | 59.23 | 37.00 |
| 32.30 | 7.10 | 78.02 | 60.00 |
| 30.30 | 7.10 | 76.57 | 37.00 |
| 19.00 | 7.60 | 60.00 | 60.00 |
| 42.00 | 19.20 | 54.29 | 50.00 |
| 60.00 | 50.00 | 16.67 | 25.00 |
| 28.00 | 9.20 | 67.14 | 33.00 |
| 23.00 | 8.10 | 64.78 | 36.00 |
| 15.00 | 8.50 | 43.33 | 30.20 |
| 60.00 | 11.80 | 80.33 | 38.00 |
| 40.30 | 22.40 | 44.42 | 36.00 |
| 57.00 | 8.50 | 85.09 | |
| 37.00 | 7.10 | 80.81 | |
| 60.00 | 60.00 | 0.00 | |
| 37.00 | 6.60 | 82.16 | |
| 18.00 | 8.00 | 55.56 | |
| 60.00 | 60.00 | 0.00 | |
| 50.00 | 9.30 | 81.40 | |
| 51.00 | 39.00 | 23.53 | |
| 25.00 | 7.40 | 70.40 | |
| 33.00 | 15.00 | 54.55 | |
| 36.00 | 5.00 | 86.11 | |

| | | |
|---|---|---|
| 30.20 | 5.30 | 82.45 |
| 38.00 | 6.90 | 81.84 |
| 36.70 | 12.00 | 67.30 |
| 36.00 | 6.70 | 81.39 |

| IOP M 1 after UCP | IOP reduction M 1 after UCP(%) | Pre-UCP IOP matched M 3 afer UCP | IOP M 3 after UCP |
|---|---|---|---|
| 26.30 | 56.17 | 60.00 | 38.00 |
| 37.40 | 37.67 | 56.20 | 41.40 |
| 15.20 | 74.67 | 60.00 | 25.40 |
| 8.60 | 65.04 | 40.80 | 8.40 |
| 16.10 | 60.54 | 24.60 | 10.70 |
| 7.10 | 83.22 | 60.00 | 18.00 |
| 7.50 | 69.51 | 41.20 | 24.60 |
| 7.40 | 80.98 | 30.90 | 7.50 |
| 18.20 | 69.67 | 23.30 | 22.20 |
| 18.40 | 55.34 | 33.30 | 19.80 |
| 7.00 | 77.35 | 37.00 | 12.10 |
| 24.10 | 57.42 | 41.10 | 12.70 |
| 22.80 | 2.15 | 51.50 | 45.00 |
| 18.10 | 45.65 | 17.30 | 13.90 |
| 45.00 | 12.62 | 18.00 | 14.40 |
| 13.00 | 24.86 | 41.00 | 14.50 |
| 8.70 | 51.67 | 17.30 | 14.40 |
| 12.90 | 68.54 | 26.00 | 47.00 |
| 9.50 | 45.09 | 32.30 | 25.00 |
| 19.00 | 5.00 | 30.30 | 10.00 |
| 22.10 | 15.00 | 19.00 | 13.10 |
| 7.10 | 78.02 | 42.00 | 7.70 |
| 7.10 | 76.57 | 28.00 | 35.30 |
| 5.90 | 68.95 | 40.30 | 5.00 |
| 17.20 | 59.05 | 57.00 | 13.70 |
| 9.10 | 60.43 | 37.00 | 11.00 |
| 10.90 | 27.33 | 60.00 | 6.70 |
| 6.50 | 83.87 | 37.00 | 42.00 |
| 7.80 | 86.32 | 50.00 | 7.70 |
| 18.00 | 51.35 | 25.00 | 12.30 |
| 12.00 | 80.00 | 36.00 | 19.30 |
| 13.80 | 62.70 | 36.00 | 14.30 |
| 60.00 | 0.00 | | |
| 15.00 | 70.00 | | |
| 9.70 | 61.20 | | |
| 13.00 | 60.61 | | |
| 14.00 | 61.11 | | |
| 35.00 | -15.89 | | |
| 9.80 | 74.21 | | |
| 9.00 | 75.00 | | |

| IOP reduction M 3 after UCP(%) | Pre-UCP IOP matched M 6 afer UCP | IOP M 6 after UCP |
|---|---|---|
| 36.67 | 60.00 | 18.90 |
| 26.33 | 56.20 | 46.10 |
| 57.67 | 60.00 | 28.70 |
| 79.41 | 60.00 | 44.80 |
| 56.50 | 24.60 | 42.30 |
| 70.00 | 42.30 | 9.90 |
| 40.29 | 24.60 | 16.00 |
| 75.73 | 41.20 | 46.10 |
| 4.72 | 30.90 | 26.70 |
| 40.54 | 23.30 | 23.80 |
| 67.30 | 33.30 | 24.80 |
| 69.10 | 37.00 | 12.10 |
| 12.62 | 41.10 | 12.70 |
| 19.65 | 51.50 | 47.00 |
| 20.00 | 17.30 | 18.00 |
| 64.63 | 18.00 | 26.40 |
| 16.76 | 41.00 | 14.50 |
| -80.77 | 17.30 | 11.90 |
| 22.60 | 20.00 | 19.00 |
| 67.00 | 26.00 | 20.90 |
| 31.05 | 32.30 | 18.00 |
| 81.67 | 30.30 | 10.00 |
| -26.07 | 19.00 | 13.10 |
| 87.59 | 42.00 | 8.90 |
| 75.96 | 28.00 | 44.20 |
| 70.27 | 40.30 | 6.00 |
| 88.83 | 57.00 | 13.70 |
| -13.51 | 37.00 | 10.90 |
| 84.60 | 60.00 | 6.70 |
| 50.80 | 37.00 | 10.00 |
| 46.39 | 50.00 | 31.00 |
| 60.28 | 25.00 | 17.00 |

| IOP reduction M 6 after UCP(%) | Pre-UCP IOP matched M 12 afer UCP | IOP M 12 after UCP |
|---|---|---|
| 68.50 | 60.00 | 33.10 |
| 17.97 | 56.20 | 11.40 |
| 52.17 | 60.00 | 55.00 |
| 25.33 | 42.30 | 8.10 |
| -71.95 | 24.60 | 11.70 |
| 76.60 | 41.20 | 30.30 |
| 34.96 | 30.90 | 48.00 |
| -11.89 | 23.30 | 20.80 |
| 13.59 | 18.00 | 18.00 |
| -2.15 | 17.30 | 14.30 |
| 25.53 | 19.00 | 12.40 |
| 67.30 | 42.00 | 9.50 |
| 69.10 | 28.00 | 44.40 |
| 8.74 | 37.00 | 13.00 |
| -4.05 | | |
| -46.67 | | |
| 64.63 | | |
| 31.21 | | |
| 5.00 | | |
| 19.62 | | |
| 44.27 | | |
| 67.00 | | |
| 31.05 | | |
| 78.81 | | |
| -57.86 | | |
| 85.11 | | |
| 75.96 | | |
| 70.54 | | |
| 88.83 | | |
| 72.97 | | |
| 38.00 | | |
| 32.00 | | |

| IOP reduction M 12 after UCP(%) | Pre-UCP IOP matched M 18 afer UCP | IOP M18 after UCP | IOP reduction M18 after UCP(%) |
|---|---|---|---|
| 44.83 | 60.00 | 24.70 | 58.83 |
| 79.72 | 60.00 | 60.00 | 0.00 |
| 8.33 | 24.60 | 30.00 | -21.95 |
| 80.85 | 42.30 | 15.00 | 64.54 |
| 52.44 | 24.60 | 12.00 | 51.22 |
| 26.46 | 30.90 | 60.00 | -94.17 |
| -55.34 | 23.30 | 9.70 | 58.37 |
| 10.73 | 26.00 | 35.30 | -35.77 |
| 0.00 | 19.00 | 15.70 | 17.37 |
| 17.34 | 28.00 | 15.00 | 46.43 |
| 34.74 | | | |
| 77.38 | | | |
| -58.57 | | | |
| 64.86 | | | |

| Pre-UCP AGMs matched D1 afer UCP | AGMs D1 after UCP | Pre-UCP AGMs matched D7 afer UCP | AGMs D7 after UCP |
|---|---|---|---|
| 3.00 | 2.00 | 3.00 | 3.00 |
| 2.00 | 2.00 | 3.00 | 2.00 |
| 3.00 | 2.00 | 4.00 | 3.00 |
| 4.00 | 3.00 | 1.00 | 2.00 |
| 1.00 | 2.00 | 2.00 | 2.00 |
| 2.00 | 2.00 | 2.00 | 2.00 |
| 2.00 | 2.00 | 3.00 | 3.00 |
| 3.00 | 3.00 | 3.00 | 1.00 |
| 3.00 | 3.00 | 3.00 | 2.00 |
| 3.00 | 3.00 | 2.00 | 2.00 |
| 2.00 | 2.00 | 2.00 | 1.00 |
| 2.00 | 2.00 | 3.00 | 3.00 |
| 3.00 | 3.00 | 3.00 | 3.00 |
| 3.00 | 3.00 | 2.00 | 2.00 |
| 2.00 | 2.00 | 3.00 | 1.00 |
| 3.00 | 3.00 | 4.00 | 1.00 |
| 4.00 | 2.00 | 2.00 | 3.00 |
| 2.00 | 2.00 | 2.00 | 2.00 |
| 2.00 | 2.00 | 2.00 | 2.00 |
| 2.00 | 2.00 | 3.00 | 1.00 |
| 3.00 | 3.00 | 2.00 | 2.00 |
| 2.00 | 2.00 | 2.00 | 2.00 |
| 2.00 | 2.00 | 3.00 | 1.00 |
| 3.00 | 2.00 | 3.00 | 3.00 |
| 3.00 | 3.00 | 4.00 | 3.00 |
| 4.00 | 4.00 | 2.00 | 1.00 |
| 2.00 | 2.00 | 3.00 | 0.00 |
| 3.00 | 3.00 | 2.00 | 1.00 |
| 2.00 | 2.00 | 3.00 | 1.00 |
| 3.00 | 1.00 | 1.00 | 1.00 |
| 1.00 | 1.00 | 3.00 | 0.00 |
| 3.00 | 0.00 | 3.00 | 0.00 |
| 3.00 | 0.00 | 3.00 | 2.00 |
| 3.00 | 2.00 | 4.00 | 3.00 |
| 4.00 | 3.00 | 3.00 | 3.00 |
| 3.00 | 3.00 | 3.00 | 2.00 |
| 3.00 | 3.00 | 2.00 | 0.00 |
| 3.00 | 2.00 | 3.00 | 0.00 |
| 2.00 | 1.00 | 3.00 | 0.00 |
| 2.00 | 2.00 | 3.00 | 2.00 |
| 3.00 | 3.00 | 3.00 | 2.00 |
| 3.00 | 3.00 | 2.00 | 2.00 |
| 3.00 | 3.00 | 3.00 | 3.00 |
| 3.00 | 2.00 | 4.00 | 2.00 |
| 3.00 | 2.00 | 3.00 | 0.00 |
| 2.00 | 2.00 | 4.00 | 3.00 |
| 3.00 | 3.00 | 2.00 | 0.00 |
| 4.00 | 2.00 | 4.00 | 3.00 |
| 3.00 | 3.00 | 3.00 | 1.00 |
| 4.00 | 3.00 | 3.00 | 2.00 |
| 4.00 | 2.00 | 3.00 | 2.00 |

| | | | |
|---|---|---|---|
| 4.00 | 1.00 | 3.00 | 0.00 |
| 2.00 | 0.00 | 3.00 | 3.00 |
| 4.00 | 3.00 | 4.00 | 1.00 |
| 3.00 | 1.00 | 2.00 | 2.00 |
| 3.00 | 2.00 | | |
| 3.00 | 2.00 | | |
| 3.00 | 3.00 | | |
| 3.00 | 3.00 | | |
| 4.00 | 3.00 | | |
| 2.00 | 2.00 | | |

| Pre-UCP AGMs matched M1 afer UCP | AGMs M1 after UCP | Pre-UCP AGMs matched M3 afer UCP |
|---|---|---|
| 3.00 | 3.00 | 3.00 |
| 4.00 | 3.00 | 3.00 |
| 2.00 | 2.00 | 4.00 |
| 2.00 | 1.00 | 3.00 |
| 3.00 | 0.00 | 2.00 |
| 3.00 | 0.00 | 3.00 |
| 2.00 | 0.00 | 2.00 |
| 2.00 | 0.00 | 4.00 |
| 3.00 | 3.00 | 2.00 |
| 2.00 | 2.00 | 3.00 |
| 4.00 | 0.00 | 2.00 |
| 2.00 | 3.00 | 2.00 |
| 2.00 | 1.00 | 3.00 |
| 3.00 | 3.00 | 4.00 |
| 3.00 | 3.00 | 2.00 |
| 4.00 | 3.00 | 3.00 |
| 2.00 | 0.00 | 2.00 |
| 3.00 | 0.00 | 1.00 |
| 2.00 | 1.00 | 3.00 |
| 3.00 | 1.00 | 3.00 |
| 1.00 | 1.00 | 3.00 |
| 3.00 | 0.00 | 4.00 |
| 3.00 | 0.00 | 3.00 |
| 3.00 | 1.00 | 3.00 |
| 4.00 | 3.00 | 3.00 |
| 2.00 | 0.00 | 2.00 |
| 3.00 | 0.00 | 3.00 |
| 3.00 | 3.00 | 4.00 |
| 3.00 | 0.00 | 2.00 |
| 2.00 | 0.00 | 3.00 |
| 3.00 | 3.00 | 3.00 |
| 4.00 | 0.00 | 2.00 |
| 4.00 | 3.00 | |
| 2.00 | 0.00 | |
| 3.00 | 1.00 | |
| 3.00 | 0.00 | |
| 3.00 | 0.00 | |
| 3.00 | 0.00 | |
| 3.00 | 0.00 | |
| 2.00 | 0.00 | |

| AGMs M3 after UCP | Pre-UCP AGMs matched M6 afer UCP | AGMs M6 after UCP |
|---|---|---|
| 3.00 | 3.00 | 3.00 |
| 3.00 | 3.00 | 3.00 |
| 2.00 | 4.00 | 2.00 |
| 0.00 | 2.00 | 3.00 |
| 2.00 | 2.00 | 0.00 |
| 2.00 | 3.00 | 0.00 |
| 4.00 | 2.00 | 2.00 |
| 0.00 | 2.00 | 3.00 |
| 2.00 | 4.00 | 1.00 |
| 3.00 | 2.00 | 2.00 |
| 2.00 | 3.00 | 3.00 |
| 2.00 | 2.00 | 0.00 |
| 3.00 | 2.00 | 0.00 |
| 3.00 | 3.00 | 3.00 |
| 1.00 | 4.00 | 3.00 |
| 1.00 | 2.00 | 1.00 |
| 0.00 | 3.00 | 2.00 |
| 2.00 | 2.00 | 2.00 |
| 1.00 | 3.00 | 2.00 |
| 0.00 | 1.00 | 3.00 |
| 1.00 | 3.00 | 2.00 |
| 3.00 | 3.00 | 0.00 |
| 2.00 | 3.00 | 1.00 |
| 0.00 | 4.00 | 3.00 |
| 0.00 | 3.00 | 3.00 |
| 1.00 | 3.00 | 0.00 |
| 1.00 | 3.00 | 0.00 |
| 0.00 | 2.00 | 3.00 |
| 0.00 | 3.00 | 1.00 |
| 3.00 | 4.00 | 3.00 |
| 0.00 | 2.00 | 0.00 |
| 0.00 | 3.00 | 3.00 |

| Pre-UCP AGMs matched M12 afer UCP | AGMs M12 after UCP | Pre-UCP AGMs matched M18 afer UCP | AGMs M18 afte |
|---|---|---|---|
| 3.00 | 2.00 | 3.00 | 2.00 |
| 3.00 | 2.00 | 2.00 | 3.00 |
| 2.00 | 3.00 | 2.00 | 2.00 |
| 3.00 | 0.00 | 3.00 | 0.00 |
| 2.00 | 2.00 | 2.00 | 3.00 |
| 2.00 | 3.00 | 4.00 | 1.00 |
| 4.00 | 3.00 | 2.00 | 3.00 |
| 2.00 | 3.00 | 1.00 | 3.00 |
| 2.00 | 3.00 | 3.00 | 1.00 |
| 2.00 | 3.00 | 3.00 | 3.00 |
| 3.00 | 1.00 | | |
| 4.00 | 3.00 | | |
| 3.00 | 3.00 | | |
| 2.00 | 3.00 | | |

r UCP
